# Evolution of Microstructure and Mechanical Properties of P92 Main Steam Pipelines After Long-Term Service

**DOI:** 10.3390/ma18194432

**Published:** 2025-09-23

**Authors:** Haitao Dong, Xianxi Xia, Qinzheng Ma, Yunting Lai, Xiao Jin, Baoyin Zhu

**Affiliations:** 1 Suzhou Nuclear Power Research Institute, Suzhou Xihuan Road 1688, Suzhou 215004, China; donght1984@163.com (H.D.); 15962169551@163.com (Q.M.); 15950061457@163.com (Y.L.); jusure@outlook.com (B.Z.); 2 National Engineering Research Center for Nuclear Power Plant Safety & Reliability, Suzhou 215004, China

**Keywords:** P92 martensitic steel, microstructural evolution, mechanical property degradation, creep resistance, long-term service

## Abstract

P92 martensitic heat-resistant steel is widely used in ultra-supercritical (USC) thermal power units due to its excellent creep resistance and high-temperature strength. However, prolonged exposure to high temperatures induces significant microstructural degradation, compromising mechanical properties and operational safety. This study investigates the evolution of microstructure and mechanical properties in P92 steel extracted from main steam pipelines after service durations of 30,000 h, 47,000 h, 56,000 h, 70,000 h, and 93,000 h. Comparative analyses of impact toughness, tensile strength, and creep strength were conducted and advanced characterization of SEM and TEM was used to investigate the microstructural evolution. The results reveal a progressive decline in mechanical properties with increasing service time. Specifically, impact toughness decreased by approximately 66.8%, room-temperature tensile strength reduced by 9.62%, and high-temperature tensile strength at 610 °C declined by 31.6%. Notably, the 10^5^ hour creep rupture strength exhibited a 10.4% decrease compared to as-received material. This decline is attributed to microstructural changes including precipitate coarsening, martensite lath boundary degradation, dislocation reconfiguration, and severe grain coarsening. The coarsening of precipitates weakens their bonding with the matrix, while the widening of martensite laths reduces resistance to crack propagation and dislocation movement, jointly contributing to strength deterioration.

## 1. Introduction

P92 martensitic heat-resistant steel, renowned for its exceptional creep resistance and high temperature strength, is extensively utilized in the manufacturing of main steam pipelines for ultra-supercritical (USC) thermal power units [1,2,3]. These pipelines in USC boilers must endure demanding conditions characterized by elevated temperatures, high pressures, and prolonged stable operation, necessitating stringent service life requirements of 100,000 to 200,000 h [4]. During long-term exposure to high temperatures, P92 steel undergoes significant microstructural evolution, including the coarsening and transformation of precipitates, degeneration of the martensitic lath structure, and weakening of grain boundaries accompanied by void nucleation [5,6]. These microstructural evolutions profoundly influence the strength degradation of P92 steel. Investigating the correlation between microstructural changes and strength variations in P92 steel under prolonged high-temperature conditions plays a crucial role in ensuring the safe operation of power plant components.

The main precipitated phases in P92 steel include M_23_C_6_ phase, Laves phase, and MX phase [7]. The primary strengthening mechanisms of P92 heat-resistant steel include solid solution strengthening, precipitation strengthening, substructure strengthening, and dislocation strengthening [8]. Extensive research has been conducted on the microstructural evolution of P92 steel under high-temperature aging [9,10,11,12,13,14]. Under prolonged high-temperature conditions, significant microstructural changes occur in P92 steel, such as precipitation and coarsening of secondary phases, lath structure coarsening, and dislocation recovery and annihilation [15]. Studies by Sun and Guo et al. [7,13] have demonstrated that the coarsening of Laves phases and recovery of martensitic laths during long-term aging significantly reduce the high-temperature strength of P92 steel. Research by Khayatzadeh et al. [16] further revealed that microstructural changes induced by varying aging durations accelerate void nucleation and creep crack propagation, leading to shortened creep life. These findings indicate that such microstructural evolution inevitably deteriorates high-temperature mechanical properties, compromising the operational safety of P92 components under high-pressure and high-temperature conditions. However, conventional accelerated testing methods, which simulate material degradation by elevating temperature and stress, fail to accurately reflect the performance evolution under long-term high-temperature and low-stress service conditions. Currently, experimental, research and long-term tracking data on P92 steel under actual service conditions remain insufficient both domestically and internationally. The impact of microstructural evolution on the macroscopic mechanical properties of materials under long-term service conditions has not been studied in detail yet.

This study investigates P92 martensitic heat-resistant steel extracted from the main steam pipeline of USC power plant after service durations of 30,000 h, 47,000 h, 56,000 h, 70,000 h, and 93,000 h. Comparative analyses of material properties including toughness, high-temperature tensile strength, and creep strength were conducted on the as-received and service-exposed samples. Advanced characterization techniques (SEM, TEM) were employed to elucidate the evolution of precipitates and microstructural features during service. This study aims to clarify the intrinsic microstructural degradation mechanisms of P92 steel under real service conditions and establish correlations between microstructural changes and mechanical property deterioration. These insights provide a foundation for predicting service life and optimizing material performance in a high-temperature environment.

## 2. Material and Experimental Procedures

### 2.1. Material

**Figure 1:**
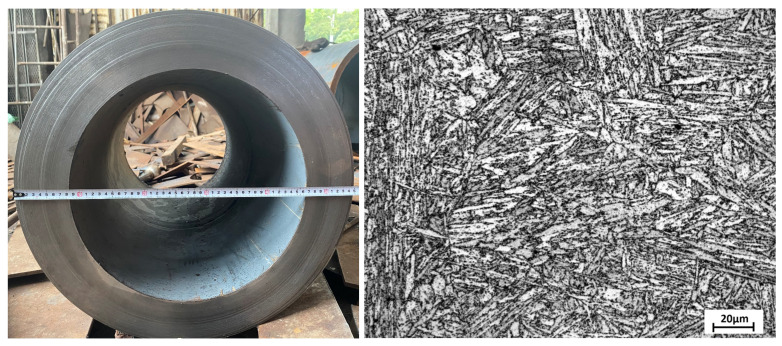
Micrograph of the as-received material.

The P92 martensitic heat-resistant steel investigated in this study was extracted from the main steam pipeline of a 1000 MW coal-fired turbine generator unit in a power plant. The unit commenced operation in 2007 and has accumulated 93,510 service hours. The pipeline operates at a temperature of 610 °C and a pressure of 26.25 MPa. The as-received microstructure of the P92 steel is shown in Figure 1. Metallographic analysis revealed that the matrix of the as-received material exhibited a typical lath martensitic microstructure, with distinct lath boundaries. Previous studies confirm that the primary precipitates in the as-received P92 steel include M_23_C_6_ carbides and MX phases [17]. Specifically, coarse M_23_C_6_ and Laves phases predominantly reside at grain boundaries or lath interfaces (dislocation-rich regions), while finer MX phases are dispersed within the matrix, with a minor fraction located at dislocation interfaces. To systematically evaluate the performance evolution of P92 steel during long-term service, samples were collected from the main steam pipeline at different service intervals: 0 h (as received), 30,000 h, 47,000 h, 56,000 h, 70,000 h and 93,000 h. The chemical compositions of the sampled P92 steel pipes at these stages are summarized in Table 1. The chemical composition of the P92 main steam sampling pipes at all service intervals complies with ASTM-A335 [18] requirements.

### 2.2. Experimental Procedure

**Figure 2:**
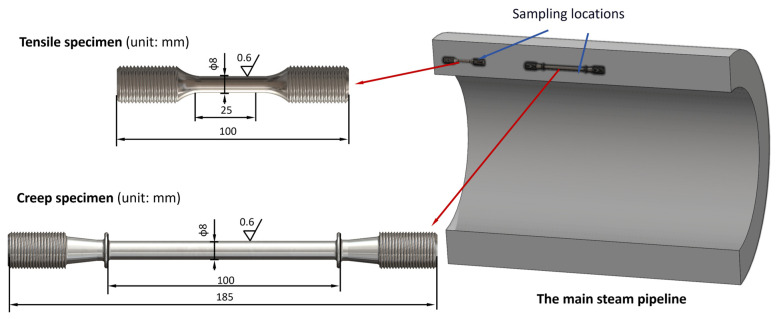
The sampling locations and specific sizes of the creep specimens and tensile specimens.

To investigate the mechanical property evolution of P92 steel during prolonged high-temperature service, systematic room-temperature (RT)/high-temperature (HT) tensile tests and creep property evaluations were conducted on specimens with varying service durations (0 h (as-received), 30,000 h, 47,000 h, 56,000 h, 70,000 h, and 93,000 h). Specimens were extracted from the middle region with testing orientations aligned along axial directions. The specific sampling locations are shown on Figure 2.

To evaluate the brittle fracture resistance of materials under high strain rates, impact tests were conducted on P92 steel specimens in different service conditions. The tests follow the GB/T 229-2020 [19] Metallic Materials—Charpy Pendulum Impact Test Method. Specimens with Charpy V-notches, measuring 10 mm × 10 mm × 55 mm, were machined and placed on the supports of the testing machine, with the ambient temperature maintained constant. After calibrating the pendulum to its initial height, it was released, and the residual kinetic energy after fracturing the specimens was converted into the fracture absorption energy.

In accordance with GB/T 228.1-2021 [20] Metallic Materials-Tensile Testing at Ambient Temperature, RT tensile tests were performed on an AG-IC Shimadzu electronic universal testing machine. Round-bar tensile specimens were employed, with sampling conducted in the longitudinal referring to GB/T 228.2-2015 [21] Metallic Materials Tensile Testing at Elevated Temperature, and HT tensile tests were carried out on an MTS-880 electro-hydraulic servo testing machine round-bar specimens (MTS Systems Corporation, Eden Prairie, MN, USA). The heating of tensile specimens was conducted in a tube furnace attached to the testing machine. Temperature was monitored and controlled using dual thermocouples: one thermocouple measured the furnace temperature, while the other was placed close to the specimen, with the latter thermocouple serving as the reference for specimen temperature control. The test temperature was set at 610 °C.

**Figure 3:**
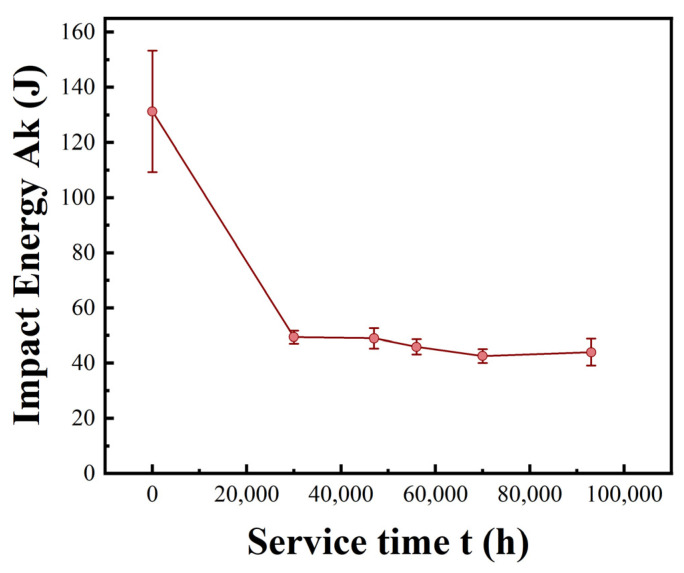
Impact energy of P92 main steam pipes under different service conditions.

To investigate the effects of long-term service conditions of main steam pipelines, creep tests were conducted on P92 steel base metal specimens from new pipes, as well as those that had been in service for 56,000 h and 93,000 h. The creep tests adhered to the GB/T 2039-2024 [22] standard for creep tensile testing and were carried out on a ZST3/3 high-temperature creep testing machine (DOLI, Munich, Germany). Considering the actual service conditions of the components, the creep test temperature was set at 610 °C. The specifications of the creep specimens are illustrated in Figure 2. The test stress levels were selected based on the results of high-temperature tensile tests and previous creep tests conducted on the pipeline. The load accuracy was maintained at ≤±1%, and the precision of creep deformation measurement was 0.001 mm. Three thermocouples were attached to the calculated gauge length of the specimens to measure temperature fluctuations and temperature gradients, ensuring a temperature control accuracy of ≤±3 °C and a temperature gradient of ≤3 °C. During the tests, the relationship between creep deformation and time was continuously measured (with measurements taken every 24 h during the steady-state phase) until the specimens ultimately fractured. The specific dimensions of the tensile specimens and creep specimens are shown in Figure 3. The detailed conditions for the tensile tests and creep tests conducted in this study are presented in Table 2 and Table 3.

### 2.3. Material Characterization

To investigate the effect of different service times on the microstructural evolution of P92 steel, metallographic, scanning electron microscopy (SEM), and transmission electron microscopy (TEM) techniques were employed in this study to analyze the microstructure of P92 steel samples subjected to varying service durations. All specimens with approximate 5 mm were extracted from each sample by wire electrical discharge machining (EDM). Initially, all specimens were subjected to preliminary grinding with sandpaper to thoroughly remove the initial surface layer. Subsequently, fine polishing was performed sequentially using polishing pastes with particle sizes of 3.5 μm, 1 μm, and 0.5 μm, with each polishing step lasting 10–15 min to ensure surface flatness. After polishing, the specimens were placed in a Bueller VibroMet 2^®^ vibratory polisher (Lake Bluff, IL, USA) for 5–8 h to effectively relieve surface residual stresses. During vibratory polishing, a polishing suspension containing approximate 60 nm SiO_2_ particles was used, which rapidly removes the slight deformation layer on the specimen surface. Each sample was measured using the high-performance backscattered electron (BSE) mode in a TESCAN CLARA UHR SEM (Brno, Czech Republic). TEM was utilized to observe the deterioration behavior of the laths and subgrain in P92 steel. For TEM sample preparation, a 0.4 mm thin foil was first cut from the area of interest, then ground to a thickness of 20 μm using Diamond Sandpaper and finally thinned to the required thickness for TEM observation via ion milling.

## 3. Results

### 3.1. Impact and Tensile Test

Impact toughness represents the ability of material to absorb energy and undergo plastic deformation without fracturing when subjected to sudden and high-speed loading (such as an impact force) [23]. The impact energies of P92 main steam pipelines in different service states are depicted in Figure 3. Observing the variation in impact energy with service time for the P92 main steam pipelines from the figure, an overall significant downward trend is evident. During the initial service period (0–20,000 h), the impact energy drops sharply from approximately 130 J to about 50 J. This decline may be attributed to the alterations in the microstructure, leading to a reduction in material toughness. In the mid-service period (20,000–60,000 h), the downward trend slows down, possibly indicating that the material has reached a relatively stable state or that the factors affecting toughness have diminished in their influence. During the late service period (60,000–100,000 h), the impact energy stabilizes within the range of 40–50 J, suggesting that the microstructure and properties of the material tended towards stability. The decrease in impact energy implies a reduction in material toughness. Throughout the service life, the pipeline may encounter impact conditions such as startups, shutdowns, and abrupt temperature changes. The reduction in toughness makes the pipeline more susceptible to brittle fracture under impact loading. This, in turn, diminishes the material’s capacity to absorb energy and undergo plastic deformation before fracture, thereby increasing the risk of sudden failure.

**Figure 4:**
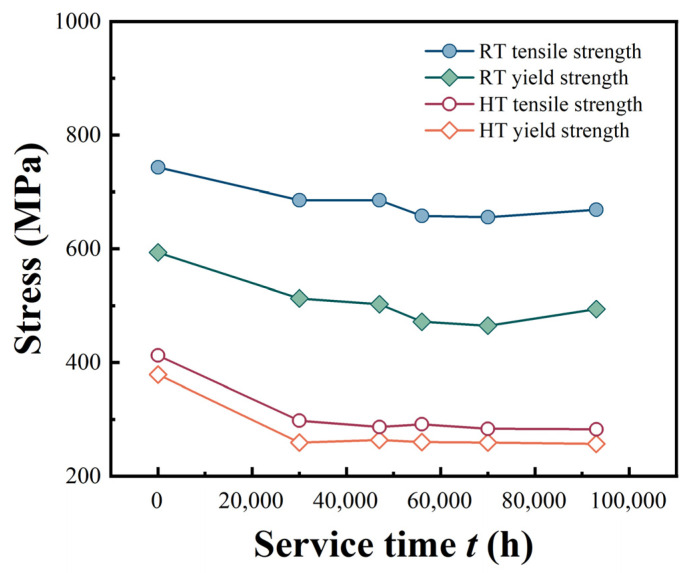
The variation in tensile properties at room/high temperature with service time.

The degradation of material properties in P92 main steam pipes during prolonged service can be comprehensively elucidated by examining the variation in tensile properties at both room temperature and elevated temperature (610 °C) as a function of service time, as depicted in Figure 4. It is apparent from the figure that both the RT tensile strength and the HT tensile strength as well as the yield strength demonstrate a decreasing tendency with prolonged service time. This unequivocally implies that the material properties of P92 main steam pipes progressively deteriorate under long-term service conditions. The microstructure of the material undergoes modifications because of continuous exposure to intricate operating conditions, including high temperature and high pressure, which leads to a continuous decline in its capacity to resist deformation and fracture under external loading. This decreasing tendency is nonlinear. It is relatively steep during the initial service phase and gradually plateaus in the later stages. In the early stages of material service, both the tensile properties and impact toughness of the material exhibit a substantial decline. Furthermore, as the material continues to be in service over time, the microstructural changes gradually achieve relatively stable conditions. During this stage, the tensile properties and impact performance of the pipeline exhibit a tendency stabilization.

### 3.2. Creep Rupture Strength and Extrapolation

Creep rupture strength is a critical parameter for evaluating long-term resistance to deformation and fracture under high-temperature and constant loading conditions [24]. In the context of pipeline actual service, the 10^5^ h creep rupture strength is of paramount importance. It serves as a key indicator of the fracture resistance of pipeline materials under long-term high temperature and constant loading conditions, directly influencing the operational lifespan and performance stability of pipelines [25]. Based on the creep test data of P92 steel at different stresses and service times, 10^5^ h creep rupture strength of the material is linearly extrapolated using the isothermal line method. At a given temperature, the empirical formula between stress and rupture time is as follows:

(1) $$t_{r}=A\sigma^{-B}$$

where *t*_r_ represents the creep rupture time, *σ* is the creep stress, and *A* and *B* are material parameters. By taking the natural logarithm of both sides of the relevant equation, and letting y=logσ, $x=logt_{r}$, $a=\frac{1}{B}\log A$, $b=-\frac{1}{B}$, the original equation can be simplified into a standard linear equation: y=a+bx. The least squares method is then employed to fit the experimental results. The extrapolated results of the rupture strength for the pipe samples are presented in Table 4. All R^2^ values exceed 0.95, indicating high reliability of the extrapolation results. This demonstrates that while short-term data extrapolation contains inherent errors, the linear model used provides statistically robust predictions for long-term creep behavior.

**Figure 5:**
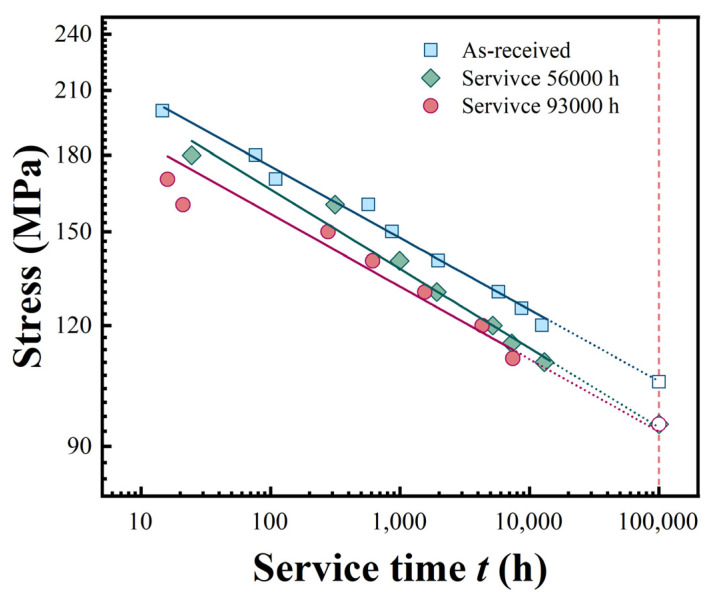
The variation in creep rupture strength with service time.

The creep test data of P92 steel at different service times and the 10^5^ h rupture strength are shown in Figure 5. Overall, each curve exhibits a trend of gradually decreasing stress with increasing time. This indicates that the creep properties of P92 steel change over time under different service conditions. The creep test data of P92 steel at various service times, along with the 10^5^ h rupture strength, are illustrated in Figure 5. Generally, each curve shows a trend of decreasing stress with increasing time. This indicates that the creep properties of P92 steel change over time under different service conditions, including new pipes and base metals after different service durations. The ability of P92 steel to resist deformation and fracture gradually weakens. Under long-term high-temperature service environments, microstructural changes occur within the material, such as carbide precipitation, coarsening and variations in dislocation density. These microstructural alterations are the main causes of material property degradation.

Comparing P92 steel in different states, notable differences are observed. The curve for the new pipelines (as received) in a relatively high-stress region indicates that the new pipe can withstand higher stresses at the same time. As the service time extends to 56,000 h and 93,000 h, the curves for the base metal gradually shift downward. This implies that the creep performance of the base metal deteriorates after service, and it can bear lower stresses at the same time. This is an inevitable consequence of microstructural changes in the base metal due to long-term high-temperature service. As the service time prolongs, the 10^5^ h rupture strength of P92 steel exhibits a gradual decline. Compared to the initial material, the 10^5^ h rupture strength experiences a 9.62% reduction after 56,000 h of service. Following 93,000 h of service, the strength demonstrates a mere 0.53% decrease compared to that at 56,000 h of service, with the magnitude of decrease being relatively smaller than that observed in the early service stage. This trend is analogous to the variation laws of impact toughness and tensile strength.

### 3.3. Microstructural Evolution Under Long-Term Conditions

**Figure 6:**
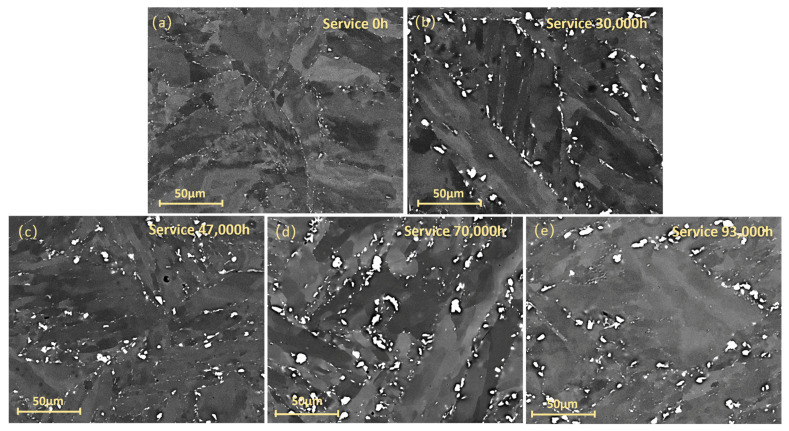
SEM images of the microstructure of P92 steel under different service conditions: (**a**) as received; (**b**) service 30,000 h; (**c**) service 47,000 h; (**d**) service 70,000 h; (**e**) service 93,000 h.

Figure 6 presents scanning electron microscopy (SEM) images illustrating the microstructure of P92 steel under long-term service conditions. Figure 6a displays the microstructure of P92 steel in the as-received state (corresponding to 0 h of service). The microstructure of the original new pipe material demonstrates a relatively uniform morphology. There are no discernible precipitate phases or microstructural anomalies present. Moreover, the grain structure and other microstructural constituents within the material are evenly distributed. As shown in Figure 6b, after 30,000 h of service, the amount of M_23_C_6_ carbides begins to increase significantly and coarsening. M_23_C_6_ carbides primarily precipitate at grain boundaries, which reduces the grain boundary cohesion and adversely affects the creep rupture strength and creep properties of the steel. Meanwhile, the Laves phase (Fe_2_(Mo, W)) starts to form gradually.

As depicted in Figure 6c, after 47,000 h of service, the M_23_C_6_ carbides continue to coarse, manifesting as larger and more irregularly shaped bright particles in the image, with a denser distribution. The quantity of the Laves phase increases, and its size further enlarges, exhibiting a more widespread distribution within the grains. After 70,000 h of service, the grain boundaries become indistinct. The M_23_C_6_ carbides grow larger and exhibit irregular shapes, while the Laves phase significantly increases in size, occupying a substantial volume within the grains. After 93,000 h of service, the microstructure undergoes severe coarsening, with the grain boundaries becoming increasingly indistinguishable. Both M_23_C_6_ carbides and the Laves phase attain significantly larger sizes and higher volume fractions. They become intertwined with each other, exerting a profoundly detrimental impact on the mechanical properties of the steel.

The evolution in the microstructure of P92 steel will have a significant impact on the mechanical properties of material [26,27]. This evolution of microstructure will reduce the strength and toughness of material and increase the likelihood of creep deformation and fracture. The growth and aggregation of precipitate phases can weaken the bonding at the precipitate–matrix interfaces, making it easier for micro cracks to form and propagate. Consequently, this results in a diminished capacity of the material to withstand external forces and resist deformation when subjected to high-temperature and long-term loading conditions.

**Figure 7:**
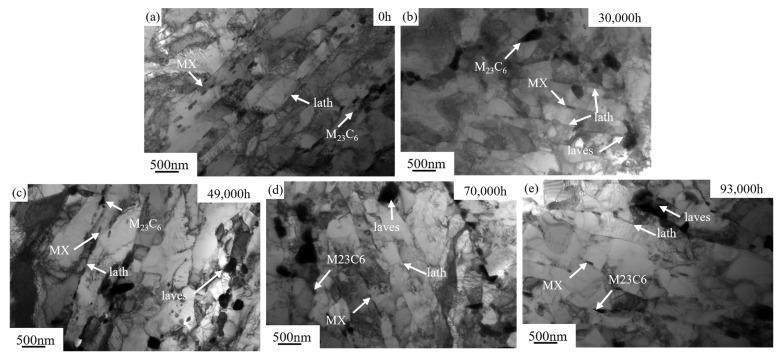
TEM images of the microstructure of P92 steel under different service conditions: (**a**) service 0 h; (**b**) service 30,000 h; (**c**) service 47,000 h; (**d**) service 70,000 h; (**e**) service 93,000 h.

TEM can achieve high-resolution imaging and perform diffraction analysis to uncover crucial microstructural features of P92 steel [28,29]. The TEM image of the original new P92 steel pipe (Figure 7a) displays a well-defined martensite lath structure with distinct lath boundaries. The precipitate phases, mainly fine M_23_C_6_ carbides and MX carbonitrides, are uniformly distributed and of small size. After 30,000 h of service (Figure 7b) martensite lath showing signs of merging or coarsening. The dislocation density increases, leading to the formation of dislocation tangles and dislocation cell structures. The M_23_C_6_ carbides significantly coarsening. At the same time, the Laves phase precipitates at grain boundaries. After 49,000 h of service (Figure 7c), the martensite lath structure undergoes further transformations with notable coarsening. Dislocation entanglement intensifies, resulting in the formation of numerous dislocation cells and subgrain structures. The M_23_C_6_ carbides exhibit significant coarsening and distribution becomes less uniform. Additionally, the Laves phase exhibit significant coarsening.

After 70,000 h of service (Figure 7d), the martensite lath width continues to increase. The dislocation structure becomes increasingly complex, with a proliferation of dislocation cells and subgrain boundaries. The M_23_C_6_ carbides undergo further coarsening and display a non-uniform distribution. The Laves phase continues to grow and undergoes coarsening. The coarsening of the Laves phase can have a significant impact on the mechanical properties of the steel. It can act as a stress concentrator, reducing the toughness and ductility of P92steel. After 93,000 h (Figure 7e) of service, pronounced grain coarsening is observed. The martensite lath structure undergoes further coarsening, and a substantial number of recrystallized grains emerge. The overall dislocation density experiences a notable reduction. The M_23_C_6_ carbides exhibit severe coarsening, and many of them may have dissolved or undergone phase transformation. The MX carbonitrides may also be affected, with some dissolving or transforming as well.

Generally, during prolonged high-temperature service, the microstructure of P92 steel experiences substantial evolution. This evolution is primarily marked by the Laves phase nucleation, M_23_C_6_ carbides, and MX phase growth and aggregation. These microstructural changes exert a direct influence on the mechanical properties of the material, including strength, toughness, and creep resistance. As the service duration extends, there is a continuous alteration in both the size and quantity of the M_23_C_6_ carbides and MX phases. This progressive change results in a gradual degradation of the material’s properties, thereby elevating the risk of material failure during operation.

## 4. Discussion

**Figure 8:**
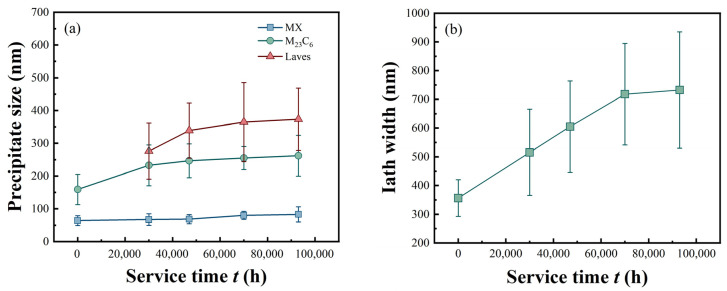
Evolution of microstructural characteristics with service time: (**a**) precipitate phases size; (**b**) martensite laths width.

The performance changes in P92 steel under long-term service conditions are closely related to the evolution of its microstructure. To further analyze the effects of precipitate phase changes and martensite lath size on the tensile and creep properties of P92 steel, statistical analyses were conducted on the precipitate phases and martensite lath sizes under different service conditions as mentioned earlier. For each experimental condition, a minimum of 3–5 random regions were analyzed through SEM imaging, with precipitate populations exceeding 100 particles per condition to ensure adequate statistical power. To ensure the reliability and representativeness of microstructural characterization results, quantitative analysis was conducted using ImageJ1.54 software with automated particle measurement (circularity range: 0.5–1.0). For each specimen, five random regions were selected for analysis, with the total number of measured particles exceeding 100 per condition. Dimensional data were presented as mean values ± standard deviation. The results are shown on Figure 8 [9]. Through statistical analysis of precipitate phase sizes, it was found that for the inspected new P92 pipes, the main precipitate phases are M_23_C_6_ and MX phases. After long-term service, the Laves phase in the P92 heat-resistant steel matrix rapidly precipitates and coarsens during the initial stage of service. After 50,000 h of service, the coarsening rate of the Laves phase gradually stabilizes. The M_23_C_6_ phase also coarsens rapidly in the early stage of service. After 30,000 h of service, the coarsening rate of the M_23_C_6_ phase gradually stabilizes. From a comparative analysis of precipitate phase sizes, the average size of the M_23_C_6_ phase is greater than that of the Laves phase, which in turn is greater than the average size of the MX phase. Regarding the comparative analysis of martensite lath sizes, after long-term service, the martensite laths continuously coarsen. Their average size gradually increases from the initial 300–400 nm in the new pipe state to 500–900 nm.

As clearly shown in Figure 3, the impact energy of P92 steel exhibits a significant decreasing trend with increasing service time. Under impact loading, fine precipitate phases can effectively hinder dislocation motion, enabling the material to absorb more energy during deformation and thus demonstrating good impact toughness. However, when the precipitate phases coarsen, the bonding force at their interfaces with the matrix weakens. They are more prone to debonding from the matrix during impact, forming micro-crack sources. These micro cracks propagate rapidly, causing the material to fracture at a relatively low energy level, thereby reducing the impact of energy. Martensite laths are an important microstructure of P92 steel. The fine lath structure can effectively impede crack propagation. During impact, when a crack encounters a lath boundary, it deflects, consuming more energy. However, when the laths widen, the number of lath boundaries decreases. This reduces the resistance to crack propagation, allowing cracks to more easily penetrate the laths and continue to expand. As a result, the material absorbs less energy during impact, and the impact of energy decreases.

The increase in M_23_C_6_ and MX phase size may lead to changes in their ability to hinder dislocation motion [30]. During tensile deformation, dislocations need to either bypass or cut through the precipitate phases. When the precipitate phases are small, the dislocation cutting mechanism dominates. As the size increases, the bypassing mechanism gradually becomes more prominent. In Figure 4, both the RT and HT tensile strengths decrease with increasing service time. This is because as the precipitate phase size increases, the bonding force at its interface with the matrix relatively weakens, reducing its ability to hinder dislocations. This makes the material more prone to plastic deformation during tension leading to a decrease in strength.

Martensite laths are also a crucial microstructure of P92 steel, and their size changes affect the strength and toughness of material [31]. An increase in lath width means a reduction in lath boundaries, which hinders dislocation motion. During tension, fewer lath boundaries make it easier for dislocations to move, reducing the material’s strength. This is consistent with the phenomenon of decreasing tensile strength with increasing service time, as shown in Figure 4. Meanwhile, changes in lath size may also affect the toughness of material. Larger lath sizes may make it easier for cracks to propagate, reducing the material’s fracture toughness.

During creep, the size and distribution of precipitate phases have a significant impact on dislocation slip and climb. Larger precipitate phase sizes may reduce the channels for dislocation slip but also decrease their ability to hinder dislocation climb [32]. As can be seen from Figure 5, the creep properties also change with increasing service time. After long-term service, the increase in precipitate phase size may make it easier for dislocations to climb and rearrange during creep, accelerating creep deformation and reducing the creep resistance of material. The martensite lath boundaries can hinder dislocation slip and climb during creep, inhibiting creep deformation. With increasing service time, the lath width increases, and the number of lath boundaries decreases. This makes it easier for dislocations to move and rearrange during creep, accelerating creep deformation. The creep resistance decreases with increasing service time, which is related to the weakened interface hindering effect caused by the increase in martensite lath size.

The size changes in precipitate phases and martensite laths are interrelated and jointly affect the tensile and creep properties of P92 steel [11,33]. During long-term service, the coarsening of precipitate phases and the widening of martensite laths both reduce the ability to hinder dislocation motion, making the material more prone to deformation during tension and creep. The decrease in tensile properties is manifested as a reduction in strength and deterioration of plasticity, while the decrease in creep properties is manifested as an increase in creep rate and a shortening of creep life.

## 5. Conclusions

In this study, impact toughness, RT/HT tensile tests, and creep tests were conducted on P92 martensitic heat-resistant steel used for main steam pipes in power plants under long-term service conditions. The changes in material strength with service time were evaluated through creep tests. SEM and TEM were employed to characterize the microstructure of P92 steel at different service times. The variations in precipitates, martensite laths, and dislocation configurations with service time were analyzed. The influence of microstructural changes on material strength under long-term service conditions was discussed. The main conclusions are as follows:

(1) RT and HT tensile properties and creep rupture strength decrease with service time. Reduced impact energy lowers material toughness, increasing brittle fracture risk. Declining tensile and yield strength weaken resistance to deformation and fracture. Reduced creep rupture strength deteriorates long-term resistance to deformation and fracture under high temperature and constant load.
(2) Under long-term high-temperature service, P92 steel undergoes significant microstructural changes. SEM images reveal increased precipitate quantity and size, forming cluster-like or chain-like structures. TEM observations show blurred, merged, or coarsened martensite lath boundaries, reduced dislocation density, and severe grain coarsening over time.
(3) Precipitate coarsening weakens the bonding force at the precipitate–matrix interface, facilitating micro-crack formation or reducing dislocation hindrance during impact and tension, thus decreasing impact energy and tensile strength. Widening martensite laths reduce lath boundaries, lowering resistance to crack propagation and dislocation movement, decreasing impact energy and tensile strength, accelerating creep deformation, and reducing creep resistance. Precipitate and martensite lath size changes are interrelated and jointly affect tensile and creep properties.
(4) In the process of pipeline design, it is essential to thoroughly account for the disparities in creep properties to ascertain the operational stress and temperature for ensuring long-term safe functioning. Enhanced service inspections and monitoring should be implemented to swiftly identify and rectify potential creep-induced damage, thereby prolonging the service life of the pipeline and guaranteeing the stable operation of the system.

## Figures and Tables

**Table 1:** Chemical composition (wt.%) of P92 steel.

	C	S	P	Si	Mn	Cr	Ni	Mo	W	B	V	Nb
0 h	0.10	0.0033	0.018	0.34	0.42	8.93	0.38	0.38	1.85	0.0012	0.17	0.067
30,000 h	0.10	0.001	0.018	0.33	0.42	8.86	0.34	0.34	1.51	0.0024	0.19	0.076
49,000 h	0.11	0.002	0.010	0.32	0.53	8.95	0.40	0.44	1.67	0.0019	0.21	0.057
70,000 h	0.094	0.014	0.014	0.26	0.47	8.68	0.37	0.41	1.58	0.0021	0.17	0.053
90,000 h	0.093	0.011	0.011	0.26	0.47	8.86	0.18	0.36	1.72	0.0015	0.16	0.064
ASME A335	0.07–0.13	≤0.01	≤0.02	≤0.5	0.3–0.6	8.5–9.5	≤0.4	0.30–0.60	1.5–2.0	0.001–0.006	0.15–0.25	0.03–0.07

**Table 2:** Tensile test parameters of the P92 steel under different service times.

Tests	Service Time *t*/h	Temperature *T*/°C	Yield Strength *σ*_s_/MPa	Tensile Strength *σ*_t_/MPa
RT	0	25	593.36	743.13
	30,000	25	512.95	685.59
	47,000	25	502.70	685.59
	56,000	25	471.96	657.99
	70,000	25	464.87	655.63
	93,000	25	494.03	669.03
HT	0	610	378.94	412.26
	30,000	610	259.12	297.75
	47,000	610	263.85	286.71
	56,000	610	259.91	291.44
	70,000	610	259.12	283.56
	93,000	610	256.76	282.72

**Table 3:** Creep test parameters of the P92 steel under different service conditions.

Material Type	Service Time *t*/h	Temperature *T*/°C	Stress σ/MPa
P92 steel	0	610	200~120
	56,000	610	200~110
	93,000	610	170~110

**Table 4:** The extrapolated results of the rupture strength of the P92 steel under different service conditions.

Material Type	Service Time *t*/h	*a*	*b*	*A*	*B*	10^5^ h Rupture Strength MPa	Coefficient of Determination R^2^
P92 steel	0	2.392	−0.074	0.028	13.51	105	0.993
	56,000	2.384	−0.082	0.0279	12.19	94.9	0.982
	93,000	2.323	−0.0697	0.0271	14.34	94.4	0.958

## Data Availability

The original contributions presented in this study are included in the article. Further inquiries can be directed to the corresponding authors.
